# Rising incidence of Kawasaki disease in Chile: analysis of national discharge databases between 2001 and 2007

**DOI:** 10.1186/1546-0096-10-S1-A90

**Published:** 2012-07-13

**Authors:** Arturo Borzutzky, Rodrigo Hoyos-Bachiloglu, Jaime Cerda, Eduardo Talesnik

**Affiliations:** 1 Pontificia Universidad Catolica de Chile, Santiago, RM, Chile

## Purpose

Incidence of Kawasaki disease (KD) varies geographically, with higher rates in East Asia and comparatively lower rates in Europe and the United States. Population-based epidemiologic studies of KD in Latin American countries have not been done. The purpose of this study is to determine incidence and demographic characteristics of KD in Chile.

## Methods

We performed a retrospective review of national hospital discharge databases, a population-based registry conducted by the Chilean Ministry of Health. We examined hospital discharges for KD (ICD10 code M30.3) between 2001 and 2007 for patients younger than 18 years of age. Hospitalization and incidence rates were calculated per 100,000 children younger than 5 years.

## Results

Seven hundred eighty-six hospitalizations attributable to KD were identified between 2001 and 2007, representing 0.03% of hospitalizations in children younger than 18 years. Median age of diagnosis was 1 year (range 0-17). Twenty-five percent of patients were younger than 1 year, 60% were between 1 and 4 years, 11% between 5 and 9 years, and 4% between 10 and 17 years. Male to female ratio was 1.6:1. Median length of hospital stay was 5 days (range 1-152). Highest hospitalization rates occurred in late winter and spring (August-November) with a smaller peak in summer (February-March). Sixty percent of patients had public health insurance and 30% of patients had private health insurance, which was over-represented among KD patients. Fifty-two percent of cases were hospitalized in the Metropolitan Region (Santiago), which had the highest KD hospitalization rate in the country. Hospitalization rate attributable to KD among children younger than 5 years was 7.6 (95% CI 7.1-7.9). Hospitalization rates significantly increased from the 2001-2004 period to the 2005-2007 period: 6.3 (95% CI 5.9-6.6) to 9.3 (95% CI 8.4-10.4), (P < 0.001). Estimated incidence of KD, assuming 10% readmission rates, was 5.7 (95% CI 5.4-6.1) per 100,000 children younger than five years for the 2001-2004 period and 8.4 (95% CI 7.6-9.6) for the 2005-2007 period (P < 0.001). Two patients died during a hospitalization attributable to KD, resulting in an acute lethality rate of 0.25%.

## Conclusion

Demographic characteristics of Chilean patients with KD are similar to what has been reported internationally. There has been an increase in hospitalization rates and estimated incidence of KD in Chile between 2001 and 2007. This may reflect a true increase in cases or improved awareness.

## Disclosure

Arturo Borzutzky: None; Rodrigo Hoyos-Bachiloglu: None; Jaime Cerda: None; Eduardo Talesnik: None.

